# Hydroxytyrosol Alleviated Hypoxia-Mediated PC12 Cell Damage through Activating PI3K/AKT/mTOR-HIF-1*α* Signaling

**DOI:** 10.1155/2022/8673728

**Published:** 2022-06-03

**Authors:** Xiaolin Li, Xiuyu Tian, Tianlong Liu, Maoxing Li, Weigang Wang, Peng Wang, Ziliang Guo

**Affiliations:** ^1^Department of Clinical Pharmacy, The 940th Hospital of Joint Logistic Support Force of Chinese People's Liberation Army, Lanzhou 730050, China; ^2^Gansu Plateau Pharmaceutical Technology Center, Lanzhou 730050, China; ^3^College of Pharmacy, Gansu University of Chinese Medicine, Lanzhou 730000, China; ^4^College of Pharmacy, Lanzhou University, Lanzhou 730000, China; ^5^Institute of Chemical Technology, Northwest Minzu University, Lanzhou 730030, China

## Abstract

**Background:**

Hypoxia exerts pressure on cells and organisms, and this pressure can occur under both pathological and nonpathological conditions. There are many reports confirmed that hydroxytyrosol has good in vitro antioxidant activity, while the research about hydroxytyrosol on hypoxia-mediated cell damage is still unclear.

**Purpose:**

The aim of this study was to investigate the effect and mechanism of hydroxytyrosol on hypoxia-mediated cell damage.

**Methods:**

We studied the effects of hydroxytyrosol on the content of reactive oxygen species, the change of antioxidant enzymes activity of SOD, CAT, and GSH-Px and the content of oxidation products MDA and GSH, and the changes of cell membrane potential and effect on PI3K/AKT/mTOR-HIF-1*α* signaling pathway under hypoxia-mediated PC12 cell.

**Results:**

PC12 cell treated with hydroxytyrosol abated the cell apoptosis and alleviated the oxidative stress through scavenging of reactive oxygen species, improving the enzyme activity and changing the content of oxidation products and alleviating mitochondria damage. Western blotting confirmed that the mechanism maybe related to the PI3K/AKT/mTOR-HIF-1*α* signaling pathway. The inhibition experiment confirmed that hydroxytyrosol takes part in the expression of protein PI3K and p-mTOR.

**Conclusion:**

Hydroxytyrosol reduced the oxidative stress and resisted the inhibition of PI3K/AKT/mTOR-HIF-1*α* signaling pathway caused by hypoxia, improved cell apoptosis, and ameliorated the antihypoxia ability of PC12 cells under hypoxia.

## 1. Introduction

Oxygen is an important part of life activities, and oxygen supply and diffusion into tissues are essential for survival. Certain organs, such as the brain and heart, have strong aerobic exercise capacity and are sensitive to hypoxia, while other organs, such as skeletal muscle, are more tolerant of prolonged hypoxia [1, 2]. The cells have evolved complex regulatory mechanisms, including regulation at the genome level to adapt to the oxygen demand [3, 4]. However, due to environmental problems (such as high altitudes areas) or own physical reasons (suffering from respiratory diseases), among others, hypoxia will still occur in different groups of people, thereby affecting various physical functions.

The PI3K/AKT/mTOR signaling pathway is a classic pathway that activates mTOR and its downstream target proteins [5]. The phosphatidylinositol 3-kinase (PI3K) signaling pathway is involved in the regulation of multiple functions such as proliferation, differentiation, apoptosis, and nuclear glucose transport. Protein kinase B (AKT), as a downstream protein of PI3K, can be activated and regulated by PI3K protein, and phosphorylation of AKT protein can promote the expression of mTOR protein. The HIF-1*α* transcription factor was first identified based on its ability to activate the erythropoietin gene in response to hypoxia. Since then, it has been shown to be activated by hypoxia in many cells and tissues. Under normoxia circumstances, the content of HIF-1 is less due to degradation. When the partial pressure of cell oxygen decreases or is affected by other regulatory factors, the degradation of HIF-1*α* decreases, and it accumulates and activates in the cytoplasm. The nucleus binds to HIF-1*α*, thereby increasing its expression. At the same time, there are related reports in the literature that mTOR protein subunit mTORC1 can regulate HIF-1*α* [6–9].

Hydroxytyrosol is a phenolic phytochemical with antioxidant properties in vitro. It is one of the important sources of natural antioxidants mainly found in olive oil and olive leaves [10]. The ability of hydroxytyrosol eliminating of free radicals is through the formation of stable hydrogen bonds with the phenoxy group through the electron-donating ability of the ortho hydroxyl group to reduce the generation of free radicals [11]. Martín et al. studied the free radical scavenging activity and antioxidant activity of hydroxytyrosol, and the results showed that hydroxytyrosol can effectively scavenge free radicals [12]. Schaffer and Halliwell found that hydroxytyrosol also has a good protective effect on low-density lipoprotein oxidation [13]. Peng et al. found that hydroxytyrosol, as a free radical scavenger and Nrf2 activator, has dual neuroprotective and cellular antioxidant defense functions [14]. Research by Zrelli et al. found that hydroxytyrosol can play an antioxidant effect and protect vascular endothelial cells by activating the Nrf2 signaling pathway and HO-1 induction [15]. In addition, hydroxytyrosol also has anti-inflammatory, antiviral, antibacterial, antifungal, and other functions, relieves cardiovascular diseases, and inhibits neurodegenerative diseases and other pharmacological effects [16].

Though the antioxidant activity of hydroxytyrosol has been studied widely, the research on its protective effect on cell damage caused by hypoxia and its mechanisms is still unclear. This article was aimed at exploring the protective effect of hydroxytyrosol on PC12 cell damage caused by hypoxia.

## 2. Materials and Methods

### 2.1. Cell Culture and Treatment

#### 2.1.1. Normoxia Cell Culture

PC12 pheochromocytoma cells were cultured in F-12k medium supplemented with 2% (*v*/*v*) FBS, 8% HS, and 1% penicillin/streptomycin at 37°C in an incubator (5% CO_2_).

#### 2.1.2. Hypoxia Cell Culture

PC12 pheochromocytoma cells were cultured in F-12 k medium supplemented with 2% (*v*/*v*) FBS, 8% HS, and 1% penicillin/streptomycin at 37°C in an incubator (5% CO_2_, 1%O_2_).

Hydroxytyrosol was bought from Chengdu Biopurify Phytochemicals Ltd. (purity > 98%, CAS: 10597-60-1). All experiments are divided into 5 groups including normoxia, hypoxia, hydroxytyrosol low (HT-L), medium (HT-M), and high (HT-H) dose group.

### 2.2. Measurement of Cell Viability

Cell viability was assessed by a cell counting kit (CCK-8). Cells were plated in 96-well plates at a density of 7 × 10^4^ cells/well in triplicate and put in 37°C, 5% CO_2_ for 24 h. Then, these cells were incubated with different concentrations of hydroxytyrosol (0 (hypoxia), 25 *μ*mol/L (HT-L), 50 *μ*mol/L (HT-M), and 100 *μ*mol/L (HT-H), respectively), at 37°C in 1%O_2_, 5% CO_2_ for 36 h. Add 10% cck8 and incubate at 37°C for 2 hours. Spectrophotometric absorbance at 450 nm was measured using a microplate reader (Thermo Scientific, Multiskan FC), and the percentage of surviving cells is calculated according to the following equation: viability of cells (%) = (A_sample_ − A_blank_)/(A_control_ − A_blank_) × 100. A_sample_ represents the OD value of pores with cell, drug, and CCK-8 solution. A_blank_ represents the OD value of pores with no cell or drug solution, only CCK-8 solution. A_control_ represents the OD value of pores with no drug solution, only cell and CCK-8 solution.

### 2.3. ROS Level Analysis

Fluorescence intensity of ROS was assessed by Reactive Oxygen Species Assay Kit (Solarbio, CA1410). Seed the cells in a 6-well plate. When the cells grow to about 80%, the blank group undergoes a medium exchange operation, and the administration group is given low (25 *μ*mol/L), medium (50 *μ*mol/L), and high (100 *μ*mol/L) concentrations of hydroxytyrosol, place them in hypoxic environments, and the active oxygen level measurement experiment was performed 36 hours later. Discard the cell culture medium, and incubate the DCFH-DA prepared in medium (1 : 1000) for 20 minutes. After carefully washing three times, the six-well plate was infiltrated with serum-free medium. Observe the results under a fluorescence microscope with GFP mode.

### 2.4. Measurement of Antioxidant Enzyme Activity of SOD, CAT, and GSH-Px

Intracellular SOD, CAT, GSH-Px enzyme activity determination and calculation are based on Nanjing Jiancheng Superoxide Dismutase (SOD) assay kit (WST-1 method) (A001-3-2), Catalase (CAT) assay kit (Visible light) (A007-1-1) and Glutathione Peroxidase (GSH-PX) assay kit (Colorimetric method) (A005-1-2) instructions for operation. Absorbance at corresponding wavelength was determined using a microplate reader.

Each data calculation formula is as follows:

(1) $$\begin{array}{c} \text{SOD inhibition rate}\left(\%\right)=\frac{\left(\left({\text{A}}_{\text{control}}-{\text{A}}_{\text{control blank}}\right)-\left({\text{A}}_{\text{sample}}-{\text{A}}_{\text{sample blank}}\right)\right)}{{\text{A}}_{\text{sample}}-{\text{A}}_{\text{sample blank}}}*100\% \end{array}$$

(2) $$\begin{array}{c} \text{SOD enzyme activity }\left(\text{U}/\text{mgprot}\right)=\frac{\text{SOD inhibition rate}}{50\%}*\frac{\text{Reaction system dilution factor}}{\text{Protein concentration of the sample to be tested }\left(\text{mgprot}/\text{mL}\right)} \end{array}$$

(3) $$\begin{array}{c} \text{CAT enzyme activity}\left(\text{U}/\text{mgprot}\right)=\frac{\left({\text{A}}_{\text{control}}-{\text{A}}_{\text{sample}}\right)*235.65/\left(60*\text{sampling volume}\right)}{\text{Protein concentration}\left(\text{mgprot}/\text{mL}\right)} \end{array}$$

(4) $$\begin{array}{c} \text{GSH}-\text{Px enzyme activity}=\frac{\left(\left({\text{A}}_{\text{nonenzyme tube}}-{\text{A}}_{\text{enzyme tube}}\right)/\left({\text{A}}_{\text{sample}}-{\text{A}}_{\text{blank}}\right)\right)*\text{standard tube concentration}\left(20\mu mol/\text{L}\right)*\text{dilution multiple}}{\text{Reaction time}*\left(\text{sampling volume}*\text{protein concentration}\right)}. \end{array}$$

### 2.5. Measurement of Content of GSH and MDA

Intracellular content of GSH and MDA determination and calculation are based on Nanjing Jiancheng reduced glutathione (GSH) assay kit (A006-2-1) and malondialdehyde (MDA) assay kit (TBA method) (A003-1-2). Absorbance at corresponding wavelength was determined using a microplate reader. Each data calculation formula is as follows:

(5) $$\begin{array}{c} \text{GSH content }\left(\mu mol/\text{gprot}\right)=\frac{\left(\left({\text{A}}_{\text{sample}}-{\text{A}}_{\text{blank}}\right)/\left({\text{A}}_{\text{standard}}-{\text{A}}_{\text{blank}}\right)\right)*\text{standard tube concentration }\left(20\mu mol/\text{L}\right)*\text{sample pretreatment dilution factor}}{\text{Protein concentration }\left(\text{gprot}/\text{L}\right)}, \end{array}$$

(6) $$\begin{array}{c} \text{MDA content }\left(\text{nmol}/\text{mgprot}\right)=\frac{\left(\left({\text{A}}_{\text{sample}}-{\text{A}}_{\text{control}}\right)/\left({\text{A}}_{\text{standard}}-{\text{A}}_{\text{blank}}\right)\right)*\text{standard concentration }\left(10\text{ nmol}/\text{mL}\right)}{\text{Protein concentration }\left(\text{mgprot}/\text{mL}\right)}. \end{array}$$

### 2.6. Membrane Potential Analysis

Fluorescence intensity of mitochondrial membrane potential was assessed by mitochondrial membrane potential assay kit with JC-1 (Beyotime, C2006). Seed the cells in a 6-well plate. When the cells grow to about 80%, the blank group undergoes a medium exchange operation, and the administration group is given low (HT-L, 25 *μ*mol/L), medium (HT-M, 50 *μ*mol/L), and high (HT-H, 100 *μ*mol/L) concentrations of hydroxytyrosol, place them in hypoxic environments, and the mitochondrial membrane potential level measurement experiment was performed 36 hours later. Discard the cell culture medium and incubate the JC-1 probe (1×) for 20 minutes. After carefully washing three times, the six-well plate was infiltrated with serum-free medium. Observe the results under a fluorescence microscope with GFP and Rhod model, respectively.

### 2.7. Western Blot Analysis

The cells were collected and lysed, and the total protein was obtained by centrifugation at 15000 rpm at 4°C. The protein concentration was determined by BCA protein detection kit (Solarbio, China) and uniformed. After adding loading buffer, then load samples, electrophoresis, and transfer membranes in sequence. After 5% skim milk blocked for 3-5 hours at 4°C, the membrane is cut according to the target molecule, and incubated with rabbit anti-PI3K p85 (#4292, CST), p-AKT (#4060, CST), p-mTOR (#2971, CST), p-4EBP1 (#2855, CST), p-P70S6K (#9205, CST), and HIF-1*α*(#14179, CST) antibodies, respectively, at 4°C overnight. After washing, goat anti-rabbit IgG H&L (HRP) (ZSBIO) was added and incubated for 1 h. *β*-Actin was used as an internal control. Proteins were visualized using Ultra ECL kit (Solarbio, china), and the image was exposed using gel imager (4600SF, TANON).

### 2.8. Effect of Hydroxytyrosol on PI3K and mTOR

Cells were cultured in 10 cm culture dishes. PI3K and mTOR inhibitors LY294002 (CSN pharm, 154447-36-6) and rapamycin (CSN pharm, 53123-88-9) were given separately when the cells were 80%. The experiment was divided into two groups of normoxia and hypoxia, and the effect of hydroxytyrosol on cell viability and related protein expression was investigated, respectively.

### 2.9. Statistical Analysis

Statistical analysis in this study was processed by SPSS 22.0 software. All data were firstly tested for normal distribution and homogeneity of variance and were expressed as mean ± standard deviation (SD). The statistical differences between the various groups were analyzed with one-way analysis of variance (ANOVA) following with Tukey's multiple comparison test. *P* < 0.05 was considered as statistical significance. The histogram was plotted using GraphPad Prism 7.0.

## 3. Results

### 3.1. Hydroxytyrosol Protected the Cell Viability of Hypoxia-Induced PC12 Cell Damage

**Figure 1:**
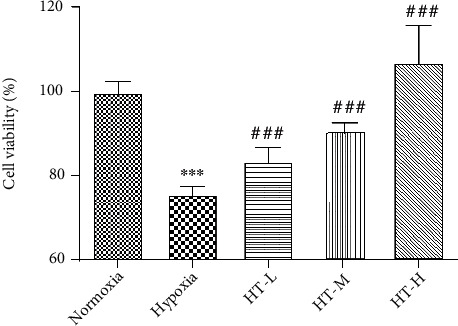
Effect of hydroxytyrosol on hypoxia-mediated PC12 cell damage. Each group represents the mean ± SD (*n* = 3). ^∗∗∗^*P* < 0.001, compared with normoxia. ^###^*P* < 0.001, compared with hypoxia.

After hypoxia for 36 hours, the viability of PC12 cells decreased significantly (*P* < 0.001). After administration of low, medium, and high concentrations of hydroxytyrosol, cell viability increased significantly at all concentrations (*P* < 0.001) and shows concentration-dependent. The result is shown in Figure 1.

### 3.2. Hydroxytyrosol Reduced the Level of Hypoxia-Induced Intracellular ROS

**Figure 2:**
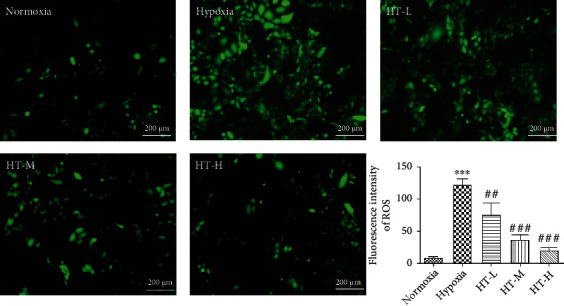
Fluorescent probe results for reactive oxygen species. Each group represents the mean ± SD (*n* = 3). ^∗∗∗^*P* < 0.001, compared with normoxia. ^##^*P* < 0.01 and ^###^*P* < 0.001, compared with hypoxia.

The underlying mechanism of the antihypoxia effect of hydroxytyrosol was investigated by measuring intracellular ROS using a fluorescent dye DCF. Compared with the control, hypoxia for 36 h alone induced a significant increase in the level of intracellular DCF-detectable ROS (*P* < 0.001), whereas hydroxytyrosol treatments reversed the level of the hypoxia-induced ROS to different degrees (Figure 2). The results indicated that hydroxytyrosol pretreatment attenuated the accumulation of hypoxia-induced ROS.

### 3.3. Hydroxytyrosol Improved the Antioxidant Enzyme Activity

**Figure 3:**
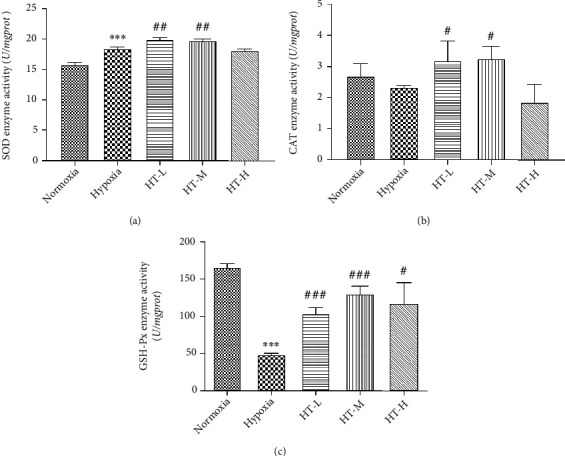
Effects of hydroxytyrosol on activities of antioxidant enzyme SOD (a), CAT (b), and GSH-Px (c) under hypoxia. Each group represents the mean ± SD (*n* = 3). ^∗∗∗^*P* < 0.001, compared with normoxia. ^#^*P* < 0.05, ^##^*P* < 0.01, and ^###^*P* < 0.001, compared with hypoxia.

The changes of antioxidant enzyme activity intracellular after administering different concentrations of hydroxytyrosol are shown in Figure 3. Compared with the normoxia group, the SOD enzyme activity of the hypoxia group was significantly increased (*P* < 0.001), and the SOD enzyme activity of the HT-L and HT-M group was significantly higher than that of the hypoxia model group (*P* < 0.01). There was no significant difference in the HT-H group (*P* > 0.05). Compared with the normoxia group, the catalase (CAT) activity of the hypoxia group was reduced, but there was no significant difference. The CAT enzyme activity of the HT-L and HT-M groups was significantly higher than that of the hypoxia group (*P* < 0.01); there was no significant difference in the high-dose group (*P* > 0.05). Compared with the normoxia group, the glutathione peroxidase (GSH-Px) activity of the hypoxia group was significantly reduced (*P* < 0.001), while the activity of GSH-Px was significantly increased after administration.

### 3.4. Hydroxytyrosol Changed the Content of MDA and GSH

**Figure 4:**
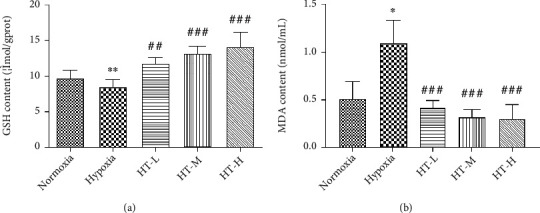
Effects of hydroxytyrosol on contents of GSH (a) and MDA (b) under hypoxia. Each group represents the mean ± SD (*n* = 3). ^∗^*P* < 0.05 and ^∗∗^*P* < 0.01, compared with normoxia. ^##^*P* < 0.01 and ^###^*P* < 0.001, compared with hypoxia.

The effects of different concentrations of hydroxytyrosol on the intracellular GSH and MDA content of PC12 cells after 36 hours of hypoxia are shown in Figure 4. Compared with the normoxia group, the intracellular glutathione (GSH) content of the hypoxia group decreased significantly (*P* < 0.01). Compared with the hypoxia group, the GSH content increased significantly after administration and has a significant concentration dependence. Compared with the normoxia group, the intracellular malondialdehyde (MDA) content of the hypoxia group increased (*P* < 0.05), and the intracellular MDA content of each concentration group was significantly lower than that of the hypoxia group after administration (*P* < 0.001) and close to the level of the normoxia group.

### 3.5. Hydroxytyrosol Raised the Membrane Potential

**Figure 5:**
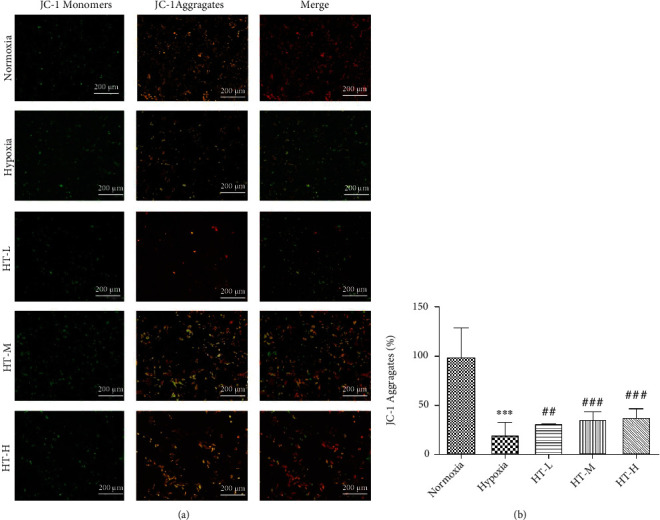
Effects of hydroxytyrosol on membrane potential under hypoxia. (a) Immunofluorescence staining for JC-1 (100×). (b) Quantitative results of the fluorescence density. Each group represents the mean ± SD (*n* = 3). ^∗∗∗^*P* < 0.001, compared with normoxia. ^##^*P* < 0.01 and ^###^*P* < 0.001, compared with hypoxia.

Compared with the normoxia group, the mitochondrial membrane potential of the hypoxia group was significantly decreased (*P* < 0.001); given different concentrations of hydroxytyrosol, the mitochondrial membrane potential was improved to varying degrees, and the increase of the mitochondrial membrane potential in the high-concentration group was improved; the effect is the most obvious (*P* < 0.001), but there is still a big difference from the normoxia group, followed by the medium concentration (*P* < 0.001). Although the effect of HT-L group is not as good as the HT-H group, the improvement effect is still statistically significant (*P* < 0.01). The results are shown in Figure 5.

### 3.6. Hydroxytyrosol Activated PI3K/AKT/mTOR-HIF-1*α* Signaling Pathway

**Figure 6:**
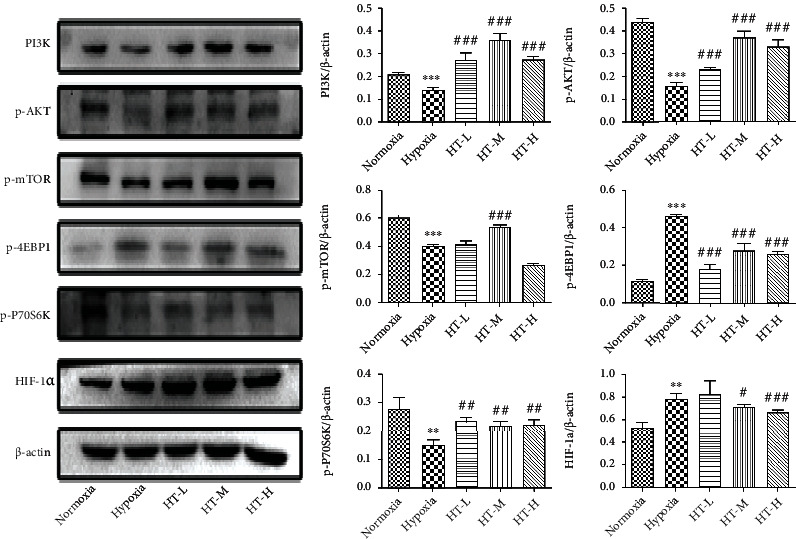
Effects of hydroxytyrosol on PI3K/AKT/mTOR-HIF-1*α* signaling pathway under hypoxia. Each group represents the mean ± SD (*n* = 3). ^∗∗^*P* < 0.01 and ^∗∗∗^*P* < 0.001, compared with normoxia. ^#^*P* < 0.05, ^##^*P* < 0.01, and ^###^*P* < 0.001, compared with hypoxia.

The result of hydroxytyrosol on hypoxia-induced PC12 cell of PI3K/AKT/mTOR-HIF-1*α* signaling pathway is shown in Figure 6. Compared with the normoxia group, the expressions of PI3K, p-AKT, and p-P70S6K all decreased significantly after hypoxia (PI3K, p-AKT: *P* < 0.001, p-P70S6K: *P* < 0.01). After given different concentrations of hydroxytyrosol, compared with the hypoxia group, the expressions of PI3K and p-AKT protein were significantly increased (*P* < 0.001), and the HT-M group had the best effect. Compared with the normoxia group, the expression of p-mTOR protein decreased significantly after hypoxia (*P* < 0.001). After the administration of different concentrations of hydroxytyrosol, the expression of p-mTOR protein increased significantly in the HT-M group compared with the hypoxia group (*P* < 0.001); there was no significant change in the HT-L and HT-H groups (*P* > 0.05). Compared with the normoxia group, after hypoxia, the expression of p-4EBP1 protein increased significantly (*P* < 0.001). After giving different concentrations of hydroxytyrosol, the expression of p-4EBP1 protein was significantly downregulated compared with the hypoxia group (*P* < 0.001). Compared with the normoxia group, the expression of HIF-1*α* protein increased significantly after hypoxia (*P* < 0.001). After the administration of different concentrations of hydroxytyrosol, there was no significant change in the HT-L group compared with the hypoxia group. The expression of HIF-1*α* protein in the HT-M and HT-H groups were significantly downregulated (HT-M: *P* < 0.05; HT-H: *P* < 0.001).

### 3.7. Mechanism of Hydroxytyrosol on PI3K/AKT/mTOR-HIF-1*α* Signaling Pathway

**Figure 7:**
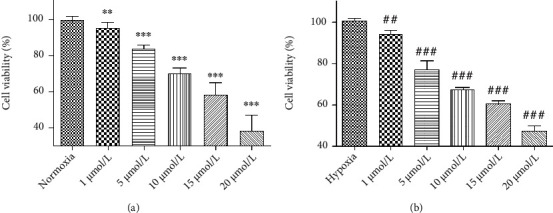
Effects of LY294002 on PC12 cell under normoxia (a) and hypoxia (b). Each group represents the mean ± SD (*n* = 3). ^∗∗^*P* < 0.01 and ^∗∗∗^*P* < 0.001, compared with normoxia. ^##^*P* < 0.01 and ^###^*P* < 0.001, compared with hypoxia.

**Figure 8:**
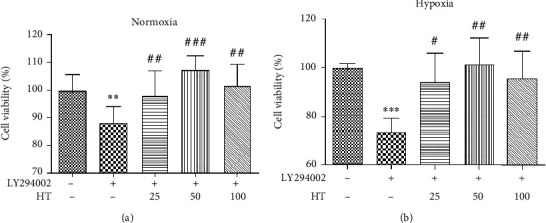
Effects of hydroxytyrosol on cell viability in LY294002 (5 *μ*mol/L) inhibited PC12 cell. (a) Normoxia condition. (b) Hypoxia condition. ^∗∗^*P* < 0.01, compared with control. ^##^*P* < 0.01 and ^###^*P* < 0.001, compared with LY294002 group.

**Figure 9:**
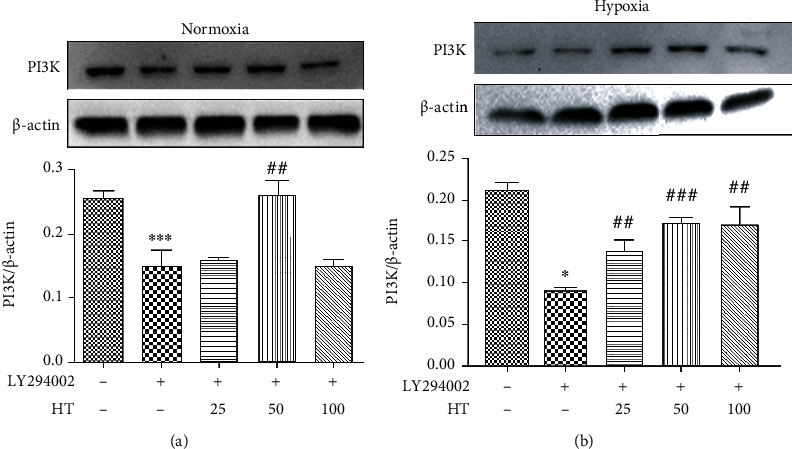
Effects of hydroxytyrosol on PI3K expression in LY294002 (5 *μ*mol/L) inhibited PC12 cell. (a) Normoxia condition. (b) Hypoxia condition. Each group represents the mean ± SD (*n* = 3). ^∗^*P* < 0.05 and ^∗∗∗^*P* < 0.001, compared with control. ^##^*P* < 0.01 and ^###^*P* < 0.001, compared with LY294002 group.

The cell viability after different concentrations of LY294002 under normoxia and hypoxia were given is shown in Figure 7. It can be seen that whether normoxia or hypoxia conditions, the cell viability is reduced with inhibitor concentration independent. When the concentration was 5 *μ*mol/L, *P* < 0.001, thus it was selected as the experimental concentration as follows. The effect of hydroxytyrosol was researched as Figure 8(a) (normoxia) and Figure 8(b) (hypoxia). It shows that hydroxytyrosol can improve the cell viability caused by PI3K inhibitor both normoxia and hypoxia. And the cell viability all recovered to the blank group. Western blot tested the protein expression of PI3K; the result is shown in Figure 9(a) (normoxia) and Figure 9(b) (hypoxia). In normoxia, the expression of PI3K protein is inhibited by LY294002, while the HT-M group of 50 *μ*mol/L hydroxytyrosol made the improvement, and the expression of PI3K protein increased significantly, low group and high group no effect. In hypoxia, the expression of PI3K protein is inhibited by LY294002, while all three groups of hydroxytyrosol made the improvement, and the effect of HT-M group is most significant.

**Figure 10:**
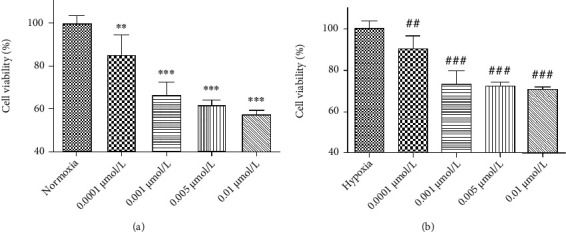
Effects of rapamycin on PC12 cell under normoxia (a) and hypoxia (b). ^∗∗^*P* < 0.01 and ^∗∗∗^*P* < 0.001, compared with normoxia. ^###^*P* < 0.001, compared with hypoxia.

**Figure 11:**
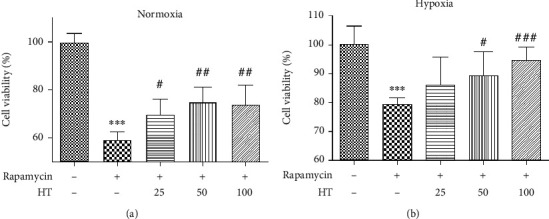
Effect of hydroxytyrosol on cell viability in rapamycin (0.001 *μ*mol/L) inhibited PC12 cell. (a) Normoxia condition. (b) Hypoxia condition. Each group represents the mean ± SD (*n* = 3). ^∗∗∗^*P* < 0.001, compared with control. ^#^*P* < 0.05, ^##^*P* < 0.01, and ^###^*P* < 0.001, compared with rapamycin group.

**Figure 12:**
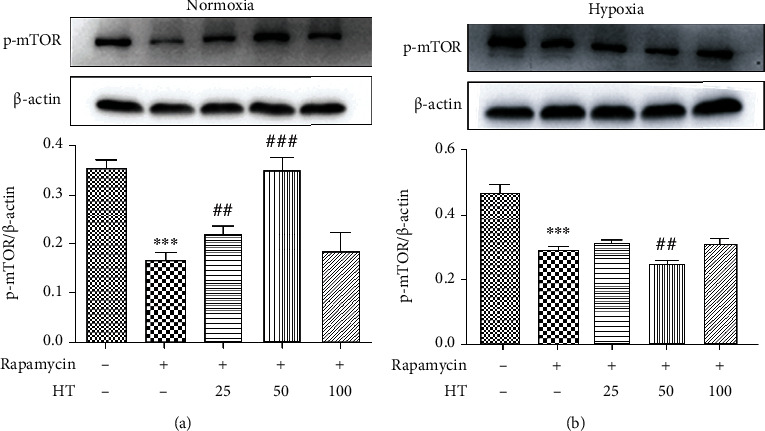
Effect of hydroxytyrosol on p-mTOR expression in rapamycin (0.001 *μ*mol/L) inhibited PC12 cell. (a) Normoxia condition. (b) Hypoxia condition. Each group represents the mean ± SD (*n* = 3). ^∗∗∗^*P* < 0.001, compared with control. ^##^*P* < 0.01 and ^###^*P* < 0.001, compared with rapamycin group.

The cell viability after different concentrations of rapamycin under normoxia and hypoxia were given is shown in Figure 10. It can be seen that whether normoxia or hypoxia conditions, the cell viability is reduced with inhibitor concentration independent. When the concentration was 0.001 *μ*mol/L, *P* < 0.001, thus it was selected as the experimental concentration as follows. The effect of hydroxytyrosol was researched as Figure 11(a) (normoxia) and Figure 11(b) (hypoxia). It shows that hydroxytyrosol can improve the cell viability caused by mTOR inhibitor both normoxia and hypoxia. And the cell viability all recovered to some degree. Western blot tested the protein expression of p-mTOR; the result is shown in Figure 12(a) (normoxia) and Figure 12(b) (hypoxia). In normoxia, the expression of p-mTOR protein is inhibited by rapamycin, while the HT-L and HT-M groups made the improvement; the expression of p-mTOR protein increased significantly, and the HT-M group made the most obvious effect; high group had no effect. In hypoxia, the expression of p-mTOR protein is inhibited by rapamycin, and all three groups of hydroxytyrosol hardly made any changes.

## 4. Discussion

Hypoxia exerts pressure on cells and organisms, and this pressure can occur under both pathological and nonpathological conditions. Hypoxia is related to diseases such as cancer, diabetes, or inflammation and also poses a challenge to people in high-altitude areas [17]. The main consumers of oxygen in cells are mitochondria. Therefore, they will be severely affected by the reduction in oxygen supply. Hypoxia changes mitochondrial fusion and fission and mitochondrial oxidative phosphorylation (OXPHOS). OXPHOS adapts to hypoxia by reshaping the electron transport chain (ETC) and the activity of the TCA cycle.

Hypoxia induces a reductive carboxylation reaction, which increases the production of ROS [18]. Excessive ROS production or insufficient antioxidants in the body can cause damage to free radicals and aerobic organisms, which is called oxidative stress [19]. Through the DCFH-DA probe-targeted experiment, after giving hydroxytyrosol, the intensity of ROS significantly eliminated, which means it does have the ability to clean the ROS produced by hypoxia.

Superoxide dismutase (SOD) is the earliest discovered ROS-metabolizing enzyme. It is an important member of the antioxidant enzyme system and is distributed in large numbers in organisms [20]. In our research, under the hypoxia circumstance, hydroxytyrosol improves the SOD enzyme activity effectively. As a marker enzyme of peroxisomes, catalase (CAT) acts by catalyzing the decomposition of hydrogen peroxide into oxygen and water [21]; after adding hydroxytyrosol and put in hypoxia, the CAT activity increased in some degrees. GSH-Px is an important peroxide decomposition enzyme whose role is to reduce toxic peroxides that exists in large quantities in the body. And its ability is mainly through catalyzing GSH to become GSSG, protecting the structure and function of cell membranes, and reducing the interference and damage of peroxides [22]. In our research, after giving hydroxytyrosol, the enzyme activity of GSH-Px increased which related to the enhanced ability of catalyzing GSH to GSSG.

GSH is a key antioxidant chemical, and its cytoplasm and mitochondrial GSH pool have the potential to buffer oxidative stress [23]. In our research, the content of GSH increased significantly which means hydroxytyrosol increased the transfer of GSH to antiperoxides. Intracellular oxygen free radicals can attack polyunsaturated fatty acids in organisms to trigger lipid peroxidation, thereby generating lipid peroxides. Thereby, MDA is often used to reflect the degree of lipid peroxidation in the body. Increased MDA means the damage of cell is caused by hypoxia, and treatment of hydroxytyrosol decreased the content of MDA.

As mentioned before, mitochondria are the main location for the production of reactive oxygen species, and mitochondrial membrane potential is one of the early features of cell apoptosis [3]. The ratio of red-green fluorescence in the cell to JC-1 probe can be used to measure the degree of mitochondrial depolarization. Hypoxia caused the change of mitochondria membrane potential, and hydroxytyrosol ameliorated the impairment.

The PI3K/AKT/mTOR signaling pathway participates in the regulation of mitochondrial function and apoptosis through a variety of mechanisms [24]. Protein mTOR is an evolutionarily conserved serine/threonine kinase that responds to a variety of stimuli, including hormones (insulin), growth factors (such as insulin-like growth factors), nutrients (amino acids), energy status, and oxygen levels to regulate cellular proliferation and growth. In order to promote cell proliferation and growth, mTOR stimulates anabolic processes, including protein synthesis, and, as recent data shows, mTOR acts as a major regulator of mitochondrial energy production. In turn, mTOR inhibits autophagy, which is a process that eliminates mitochondria. When the activity of PI3K/AKT/mTOR signal transduction is inhibited, the decrease of mTORC1 activity leads to the inhibition of its substrate proteins S6K1 and 4EBP1, which leads to the restriction of the translation of certain important proteins that regulate mitochondrial functions, such as protein synthesis and mitochondrion electron transport chain, thereby disrupting mitochondrial function [25, 26]. In this experiment, it was found that the PI3K/AKT/mTOR-HIF-1*α* signaling pathway in PC12 cells under hypoxia was inhibited, while hydroxytyrosol activated the signaling pathway. Oxidative stress is the imbalance between oxidation and antioxidation in the environment. Mitochondrial dysfunction and decreased levels of antioxidant enzymes lead to increased ROS production, which commonly causes oxidative stress and contributes to high-altitude illnesses (high-altitude pulmonary edema, high-altitude cerebral edema, etc.). Additionally, previous investigator demonstrated that PI3K/Akt/mTOR cascade was driven by ROS [27, 28]. Salidroside could suppressed LPS-induced myocardial injury by inhibiting ROS-mediated PI3K/Akt/mTOR pathway in vitro and in vivo [29]. Hydroxytyrosol's antioxidative activity was attributed to the ROS-mediated PI3K/Akt/mTOR-HIF-1*α* signaling pathway. We found that inhibition of PI3K and mTOR has been suggested to be able to regulate the PI3K/AKT/mTOR-HIF-1*α* signaling pathway by inducing oxidative stress.

Reports have shown that the hydroxytyrosol has great pharmacological potential, such as anti-inflammatory, antitumor, antiatherogenic, and antithrombotic properties [30–32]. Hydroxytyrosol also improves endothelial dysfunction, decreases oxidative stress, and has neuroprotective and cardioprotective effects [33]. These pharmacological effects are mainly achieved by improving the imbalance of the redox system and inhibiting the level of oxidative stress, thereby protecting the normal function of signal transduction (Nrf-2, NF-*κ*B, etc.) and important organelles and restoring the normal function of tissues and organs [34, 35]. Hydroxytyrosol improves mitochondrial function in hypoxic PC12 cells and reduces oxidative stress and inhibits the expression of PI3K/AKT/mTOR-HIF-1*α* signaling pathway. We believe that more conclusive studies are needed to link the potential benefits of HT with a preventive/treatment role against cardiovascular disease and to initiate new research on molecular mechanism.

## 5. Conclusions

**Figure 13:**
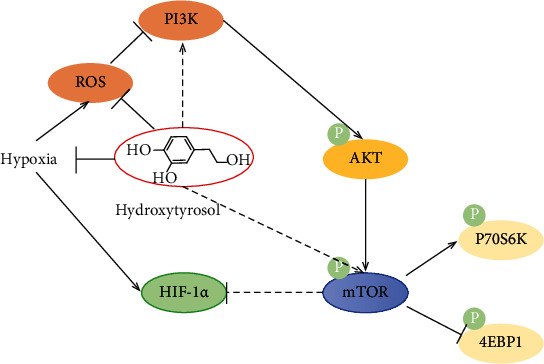
Hydroxytyrosol alleviated hypoxia-mediated PC12 cell damage through activating PI3K/AKT/mTOR-HIF-1*α* signaling pathway.

In the article, after culturing PC12 cells with hypoxia (1% O_2_, 5% CO_2_) for 36 hours, the cells were damaged by oxidative stress. After administration of hydroxytyrosol, free radicals were effectively eliminated and the degree of cell damage was alleviated. We have also found that hypoxia can inhibit the cellular PI3K/AKT/mTOR-HIF-1*α* signaling pathway, and hydroxytyrosol can effectively reduce or even reverse the inhibition. Hydroxytyrosol not only improves the activity of SOD, CAT, and GSH-Px enzymes but also increases the GSH content, reduces the content of lipid peroxidation product malondialdehyde, and effectively eliminates the reactive oxygen species produced by hypoxia in the cell. At the same time, this experiment also confirmed that hypoxia can inhibit the PI3K/AKT/mTOR-HIF-1*α* signaling pathway, and the administration of hydroxytyrosol effectively blocked the inhibition of this signaling pathway (Figure 13).

In summary, hydroxytyrosol can reduce the oxidative stress and resist the inhibition of PI3K/AKT/mTOR-HIF-1*α* signaling pathway caused by hypoxia, improve cell apoptosis, and ameliorate the antihypoxia ability of PC12 cells under hypoxia.

## Data Availability

The data sets used and analyzed in the present study are available from the corresponding authors on reasonable request.All authors have read the journal's policy on authorship agreement and conflict of interest. The authors have declared that no conflict of interest exists.
